# Analysis of causative factors and preventive strategies for traumatic orthopedic medical malpractice in Shanghai: a high-responsibility perspective

**DOI:** 10.3389/fsurg.2026.1930290

**Published:** 2026-10-30

**Authors:** Chong Chen, Hong Gao, Shiying Wang, Lei Yuan, Weixiang Xu, Hongli Yan, Xiaofei Ye, Haixia Qi, Yang Li, Yuqin Ma, Hong Wu, Ping Xu

**Affiliations:** 1 Faculty of Military Health Services, Naval Medical University (Second Military Medical University), Shanghai, China; 2 Changzheng Hospital Central Sterile Supply Department, Naval Medical University (Second Military Medical University), Shanghai, China; 3 Department of Health Care, Second Affiliated Hospital of Naval Medical University, Shanghai, China; 4 The Medical Administration and Management Department of the Shanghai Municipal Health Commission, Shanghai, China

**Keywords:** cause analysis, China, medical malpractice, preventive strategies, traumatic orthopedics

## Abstract

**Background:**

Medical malpractice is recognized as the most severe category of adverse medical events, characterized by illegality, negligent conduct, and resultant harm. Cases classified as high-responsibility-level malpractice impose substantial impacts on healthcare institutions and medical personnel. This study aims to identify negligence-related determinants contributing to such cases and to explore targeted preventive strategies designed to reduce the occurrence of high-responsibility-level medical malpractice.

**Methods:**

A total of 125 cases of traumatic orthopedic medical malpractice were retrieved from the Office of Medical Malpractice Handling under the Shanghai Municipal Health Commission. Statistical analyses were conducted using SPSS 26.0. Descriptive data were presented as counts, frequencies, and percentages. Intergroup differences in negligence-related factors between high- and low-responsibility-level categories were evaluated using chi-square tests. Additionally, binary stepwise logistic regression analysis was performed to determine independent associations between specific negligence factors and high responsibility levels.

**Results:**

Traumatic orthopedic medical malpractice cases in Shanghai demonstrated several distinct characteristics, including a relatively low mortality rate (4.00%), a notably high disability rate (74.4%), a greater occurrence in secondary hospitals (60.00%), and increased involvement of attending physicians and associate chief physicians (76.80%). High-responsibility-level cases constituted 45.60% of all included cases. Chi-square analysis indicated that inappropriate surgical procedures and violations of clinical practice standards were significantly more prevalent in the high-responsibility-level group compared with the low-responsibility-level group (*P* < 0.05). Logistic regression analysis further revealed that inappropriate surgical procedures [odds ratio (OR) = 6.078], violations of clinical practice standards (OR=5.001), and missed or misdiagnoses (OR=2.872) were independently associated with elevated responsibility levels. Stratified regression analysis further indicated that violations of clinical practice standards represented the dominant negligence factor in secondary hospitals (OR=2.94), whereas inappropriate surgical procedures constituted the primary determinant in tertiary hospitals (OR=7.20).

**Conclusions:**

Several preventive measures are recommended, including strengthening perioperative management, ensuring strict compliance with clinical diagnostic, therapeutic, and nursing protocols, standardizing imaging examinations and comprehensive physical assessments to reduce missed and misdiagnoses, enhancing physician–patient communication, standardizing medical record documentation, and reinforcing early postoperative rehabilitation strategies.

## 1 Introduction

A medical malpractice incident is defined as an event in which a healthcare institution and its medical personnel, during the provision of clinical services, are found to be in violation of healthcare administration laws and regulations, administrative statutes, departmental rules, or established diagnostic and nursing standards or protocols, thereby resulting in patient harm due to negligent conduct (1). Medical malpractice incidents are regarded as the most severe category of adverse events within medical safety management systems.

Traumatic orthopedics is recognized as one of the surgical specialties associated with the highest incidence of medical malpractice claims worldwide. The rates of malpractice and litigation within this field have remained consistently elevated on a global scale (2). A meta-analysis has estimated that the likelihood of litigation in traumatic orthopedics is approximately 23.3% (3). Data reported by the UK National Health Service (NHS) indicated that, from 2008 to 2019, a total of 8,548 litigation cases related exclusively to traumatic orthopedics were documented, with cumulative compensation exceeding 1.2 billion pounds (4). In such cases, patients are frequently subjected to irreversible adverse outcomes, including death, limb amputation, or permanent neurological impairment.

Several factors contribute to the high incidence of traumatic orthopedics in China: First, as a major manufacturing country, a relatively high incidence of traumatic injuries has been observed. Second, patients with traumatic orthopedic conditions are frequently admitted to emergency departments following traffic accidents or falls. These cases are often characterized by complex and rapidly evolving clinical presentations, frequently accompanied by visceral or even craniocerebral injuries, thereby increasing the likelihood of missed diagnoses or misdiagnoses (5–7). Third, traumatic orthopedics is a clinical specialty in which surgical intervention serves as the primary treatment modality, thereby entailing considerable operative risks and a high incidence of postoperative complications.

In medical malpractice cases, high responsibility levels refer to circumstances in which the medical party is assigned full or primary liability, representing the predominant etiological factor leading to severe outcomes such as patient death, moderate to severe disability, or damage to organs or tissues (8–10). According to the *Regulations on the Handling of Medical Accidents*, healthcare institutions involved in high-responsibility-level medical malpractice are subject to administrative sanctions ranging from formal warnings and mandated corrective suspension to revocation of operating licenses. Medical personnel may face suspension of practice or revocation of professional licensure, while the medical party is required to assume 60%–100% of financial compensation obligations. Additionally, Article 335 of the *Criminal Law of the People's Republic of China* stipulates that medical personnel who cause patient death or severe impairment of health due to gross negligence shall be sentenced to fixed-term imprisonment of not more than three years or criminal detention. It is evident that high-responsibility-level medical malpractice exerts profound adverse impacts on patients, healthcare institutions, and medical personnel alike. Therefore, effective interventions are required to address underlying negligence factors and to reduce the incidence of such cases.

Shanghai is characterized by a high concentration of medical resources, with hospitals demonstrating advanced capabilities and stringent clinical standards. Owing to well-developed transportation networks connecting surrounding regions, a pronounced metropolitan integration effect has been formed, further reinforced by the accessibility of cross-regional medical insurance settlement systems. In conjunction with substantial patient trust and long-established healthcare-seeking behaviors, the city exerts a strong siphoning effect on patients nationwide. Given the relatively high incidence of traumatic orthopedic medical malpractice cases and the sufficient availability of data, Shanghai provides a representative and robust dataset for the present study.

## 2 Materials and methods

Data for this study were obtained from the Office of Medical Malpractice Handling under the Shanghai Municipal Health Commission. A total of 125 cases of traumatic orthopedic medical malpractice occurring between January 2005 and December 2020 were collected. All cases were subjected to technical appraisal by the Shanghai Medical Association and confirmed by the Health Commission. This study was approved by the Committee on Ethics of Medicine, Naval Medical University (see certificate in the Supplementary files).

### 2.1 Data collection

Data were extracted from the aforementioned sources and categorized into the following domains: (1) baseline patient characteristics, including age, sex, trauma etiology, and initial diagnosis; (2) physician-related characteristics, including professional title, years of clinical practice, department affiliation, educational background, and institutional attributes of the hospital; and (3) incident-related variables, including the clinical course of the medical malpractice event, severity grading of malpractice, responsibility attribution based on expert appraisal, classification pathway of the malpractice case, primary medical negligence factors, and key points of dispute between physicians and patients.

During the data extraction process, all directly identifying information of patients, including names, ID numbers, hospital record numbers, and contact details, was thoroughly removed. Less sensitive demographic details, such as age, geographic region, and visit period, were generalized to minimize the risk of re-identification. All data processing was conducted entirely offline, with no connection to any public network, to ensure the protection of patient privacy.

### 2.2 Analytical methods

Data management and organization were performed using Excel 2019, and all statistical analyses were conducted with SPSS 26.0. Descriptive statistics were applied to summarize the dataset, with outcomes reported as case counts, frequencies, and proportions. Cases classified as primary responsibility or full responsibility were grouped into the high-responsibility category, whereas those classified as minor responsibility or secondary responsibility were assigned to the low-responsibility category. Intergroup differences in types of medical negligence were assessed using the chi-square (*χ*^2^) test. Furthermore, binary stepwise logistic regression analysis was conducted to determine independent associations between individual negligence factors and high responsibility level, with effect estimates reported as odds ratios (ORs) and corresponding 95% confidence intervals (CIs). A two-tailed *P* < 0.05 was defined as statistically significant. Collinearity among the independent variables was assessed using the variance inflation factor (VIF) prior to regression analysis, to rule out the influence of severe multicollinearity on the regression estimates.

Additionally, stratified analyses were performed according to hospital grade to examine whether the associations between diagnostic and therapeutic negligence factors and high responsibility level varied across institutions. The same binary stepwise logistic regression model was independently applied to secondary hospitals (*n* = 75) and tertiary hospitals (*n* = 42). Primary hospitals (*n* = 8) were excluded from stratified regression analyses due to insufficient sample size (For the classification of Chinese hospital tiers, see Supplementary Appendix 4). Variables included in these regression models and their specific assignments are summarized in Table 1.

**Table 1:** Variable assignment.

Variable	Definition and assignment
Degree of medical responsibility	Minor responsibility=1
	Secondary responsibility=2
	Primary responsibility=3
	Full responsibility=4
Missed or misdiagnosis	Present=1
	Absent=0
Improper surgery	Present=1
	Absent=0
Inadequate informed consent	Present=1
	Absent=0
Insufficient observation	Present=1
	Absent=0
Violation of diagnostic and treatment protocols	Present=1
	Absent=0
Variable	Definition and assignment
Inadequate emergency response	Present=1
	Absent=0
Other causes	Present=1
	Absent=0

## 3 Results analysis

### 3.1 Basic patient characteristics

(1) Distribution of patient sex and age

Regarding sex distribution, 83 patients (66.40%) were male, whereas 42 patients (33.60%) were female.

Regarding age distribution, individuals aged ≤20 years accounted for 6.40%, those aged 21–39 years accounted for 28.00%, those aged 40–59 years accounted for 51.20%, and those aged ≥60 years accounted for 14.40%. These findings indicate that the 40–59-year age group was associated with a higher likelihood of sustaining traumatic injuries.

(2) Distribution of trauma sources and injury sites

Trauma etiologies were distributed as follows: traffic accidents accounted for 46 cases (36.80%), fall-related injuries for 19 cases (15.20%), machinery-related injuries for 24 cases (19.20%), and daily-life injuries for 36 cases (28.80%), indicating that traffic accidents represented the predominant trauma source.

Regarding injury site distribution, extremity injuries were most frequently observed, comprising 86 cases (68.80%), followed by joint injuries in 18 cases (14.40%), spinal injuries in 13 cases (10.40%), and nerve injuries in 8 cases (6.40%). Compartment syndrome, one of the most severe complications associated with extremity injuries, was identified in 5 patients in the present case series, among whom 3 patients subsequently underwent amputation.

### 3.2 Distribution of medical negligence responsibility levels

Pursuant to the relevant provisions of the *Civil Code of the People*’*s Republic of China* and the *Regulations on the Handling of Medical Accidents*, medical malpractice responsibility levels are stratified into four categories according to the degree of negligence attributable to the healthcare institution and the extent of resultant harm: (1) Full responsibility, in which the adverse outcome is entirely attributable to negligent medical conduct; (2) Primary responsibility, in which the adverse outcome is predominantly caused by negligent medical conduct, with contributory influence from other factors; (3) Secondary responsibility, in which the adverse outcome is mainly driven by other factors, with negligent medical conduct playing a subordinate role; and (4) Minor responsibility, in which the adverse outcome is largely attributable to external factors, with only minimal contribution from negligent medical conduct.

As illustrated in Figure 1, primary responsibility and secondary responsibility constituted the dominant categories, each comprising 50 cases and jointly accounting for 80% of the total sample. By contrast, full responsibility and minor responsibility were less frequently observed, with 7 and 18 cases, respectively, together representing 20% of all cases.

**Figure 1:**
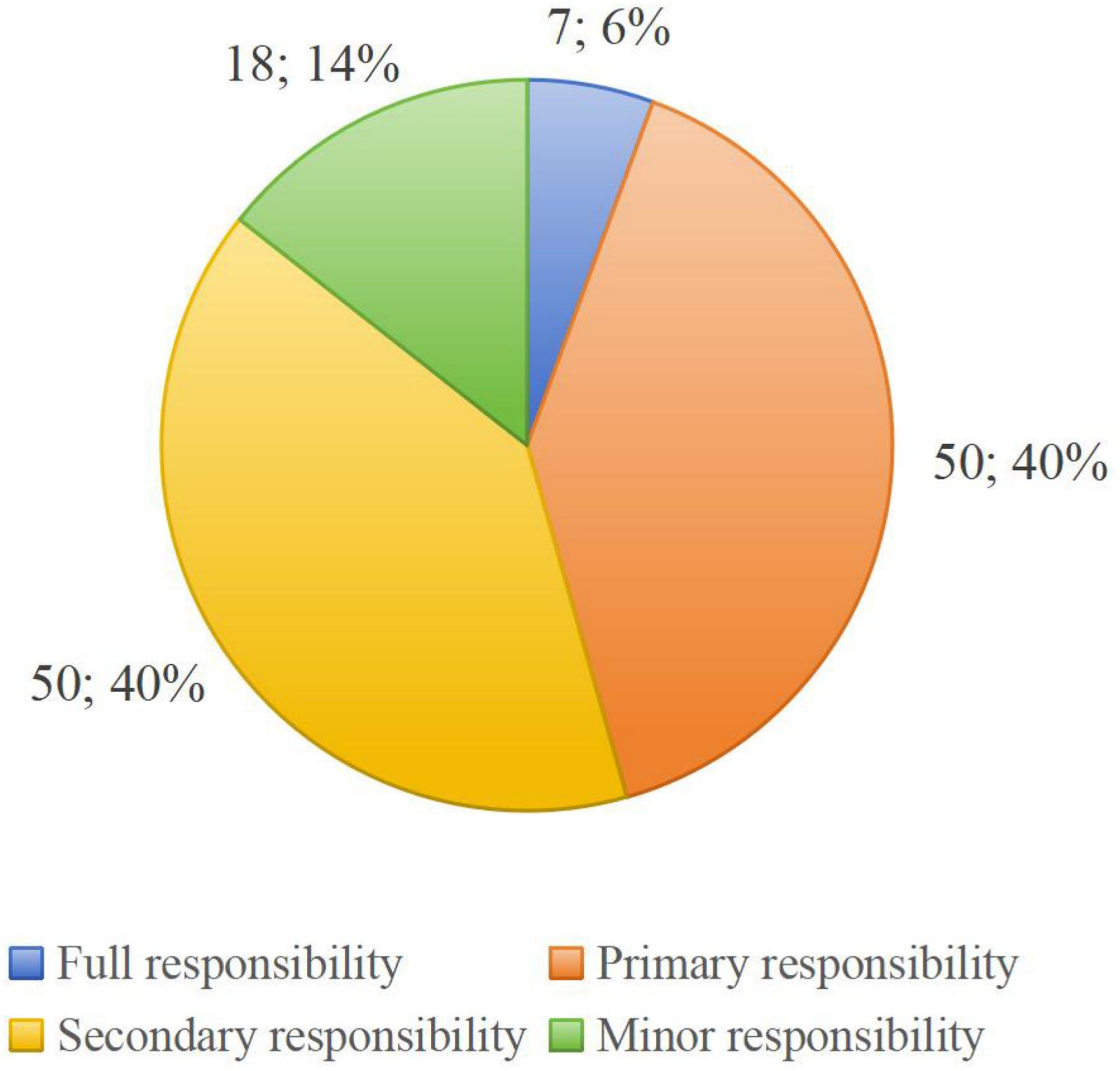
Distribution of responsibility levels among medical parties in traumatic orthopedic cases.

### 3.3 Distribution of medical malpractice grades, medical institutions, responsibility levels, and responsible parties

According to Article 4 of the Regulations on the Handling of Medical Malpractice and the Grading Standards for Medical Malpractice (Trial) (Ministry of Health Order No. 32) (11) (see Supplementary Appendix 1 for detailed classification criteria), medical malpractice in China is generally classified into four levels.

As presented in Tables 2, 3 and Figure 2, the following observations were identified.

(1) Predominance of low malpractice grades. Grade III and Grade IV malpractice cases constituted the majority of cases, with 66 and 50 cases, respectively, accounting jointly for 92.8% of the total sample. In contrast, Grade I and Grade II cases were relatively rare, with 5 and 4 cases, respectively, representing a combined proportion of 7.2%.
(2) Higher incidence of malpractice in secondary hospitals. A total of 75 cases (60.00%) of medical malpractice were recorded in secondary hospitals, compared with 42 cases (33.60%) in tertiary hospitals and 8 cases (6.40%) in primary hospitals. Several factors may account for this elevated incidence in secondary hospitals. First, under the principle of rapid and proximity-based medical access, 45.64% of patients were transported by ambulance, resulting in the majority being admitted to secondary hospitals, which are more widely distributed and more numerous. Second, a substantial gap in treatment capacity and advanced medical equipment was observed between secondary and tertiary hospitals. Third, certain cases involved violations of institutional regulations, including unauthorized patient retention and unjustified expansion of surgical indications.
(3) Disproportionate representation of associate chief physicians and attending physicians. Associate chief physicians and attending physicians accounted for 47 and 49 cases, respectively, jointly representing 76.80% of all cases, whereas chief physicians and resident physicians accounted for 12.80% and 10.40%, respectively, with a combined proportion of 23.20%. Two explanatory factors may be proposed. First, associate chief physicians and attending physicians constitute the clinical backbone across hospital levels, independently managing inpatient wards and performing surgical procedures while bearing a substantial clinical workload. However, their theoretical knowledge base, surgical proficiency, and accumulated clinical experience remain comparatively less advanced than those of chief physicians. Second, deficiencies in professional responsibility awareness and technical competency among some associate chief physicians and attending physicians may persist.
(4) Low mortality rate and high disability rate. As illustrated in Figure 2, 5 of the 125 patients (4.00%) died, indicating a relatively low mortality rate. The causes of death included septic shock with disseminated intravascular coagulation, thrombotic pulmonary embolism of the main pulmonary artery, sudden cardiac death, acute pulmonary edema, and severe upper gastrointestinal hemorrhage. All were attributable to complications rather than the primary traumatic orthopedic condition. In one representative case, a patient with a right femoral trochanteric fracture developed acute respiratory distress syndrome on postoperative day 3. The patient had a 30-year history of diabetes mellitus, and improper fluid management by the medical team contributed to acute pulmonary edema and subsequent death.

**Table 2:** Distribution of malpractice grades and responsibility levels across medical institutions of different tiers.

Category	Grade	Primary hospital (*n* = 8)	Secondary hospital (*n* = 75)	Tertiary hospital (*n* = 42)	Total (*n* = 125)
Medical malpractice grade	Grade I	0 (0.0%)	2 (2.7%)	3 (7.1%)	5 (4.0%)
	Grade II	0 (0.0%)	3 (4.0%)	1 (2.4%)	4 (3.2%)
	Grade III	4 (50.0%)	42 (56.0%)	20 (47.6%)	66 (52.8%)
	Grade IV	4 (50.0%)	28 (37.3%)	18 (42.9%)	50 (40.0%)
Responsibility level	Full responsibility	0 (0.0%)	4 (5.3%)	3 (7.1%)	7 (5.6%)
	Primary responsibility	1 (12.5%)	32 (42.7%)	17 (40.5%)	50 (40.0%)
	Secondary Responsibility	6 (75.0%)	27 (36.0%)	17 (40.5%)	50 (40.0%)
	Minor Responsibility	1 (12.5%)	12 (16.0%)	5 (11.9%)	18 (14.4%)

**Table 3:** Distribution of malpractice grades and responsibility levels among responsible parties of different titles.

Category	Grade	Chief physician (*n* = 16)	Associate chief physician (*n* = 47)	Attending physician (*n* = 49)	Resident physician (*n* = 13)	Total (*n* = 125)
Medical malpractice grade	Grade I	1 (6.25%)	2 (4.26%)	2 (4.08%)	0 (0.00%)	5 (4.00%)
	Grade II	1 (6.25%)	2 (4.26%)	1 (2.04%)	0 (0.00%)	4 (3.20%)
	Grade III	6 (37.50%)	25 (53.19%)	27 (55.10%)	8 (61.54%)	66 (52.80%)
	Grade IV	8 (50.00%)	18 (38.30%)	19 (38.78%)	5 (38.46%)	50 (40.00%)
Responsibility level	Full responsibility	0 (0.00%)	4 (8.51%)	3 (6.12%)	0 (0.00%)	7 (5.60%)
	Primary responsibility	5 (31.25%)	21 (44.68%)	19 (38.78%)	5 (38.46%)	50 (40.00%)
	Secondary Responsibility	8 (50.00%)	18 (38.30%)	18 (36.73%)	6 (46.15%)	50 (40.00%)
	Minor Responsibility	3 (18.75%)	4 (8.51%)	9 (18.37%)	2 (15.38%)	18 (14.40%)

**Figure 2:**
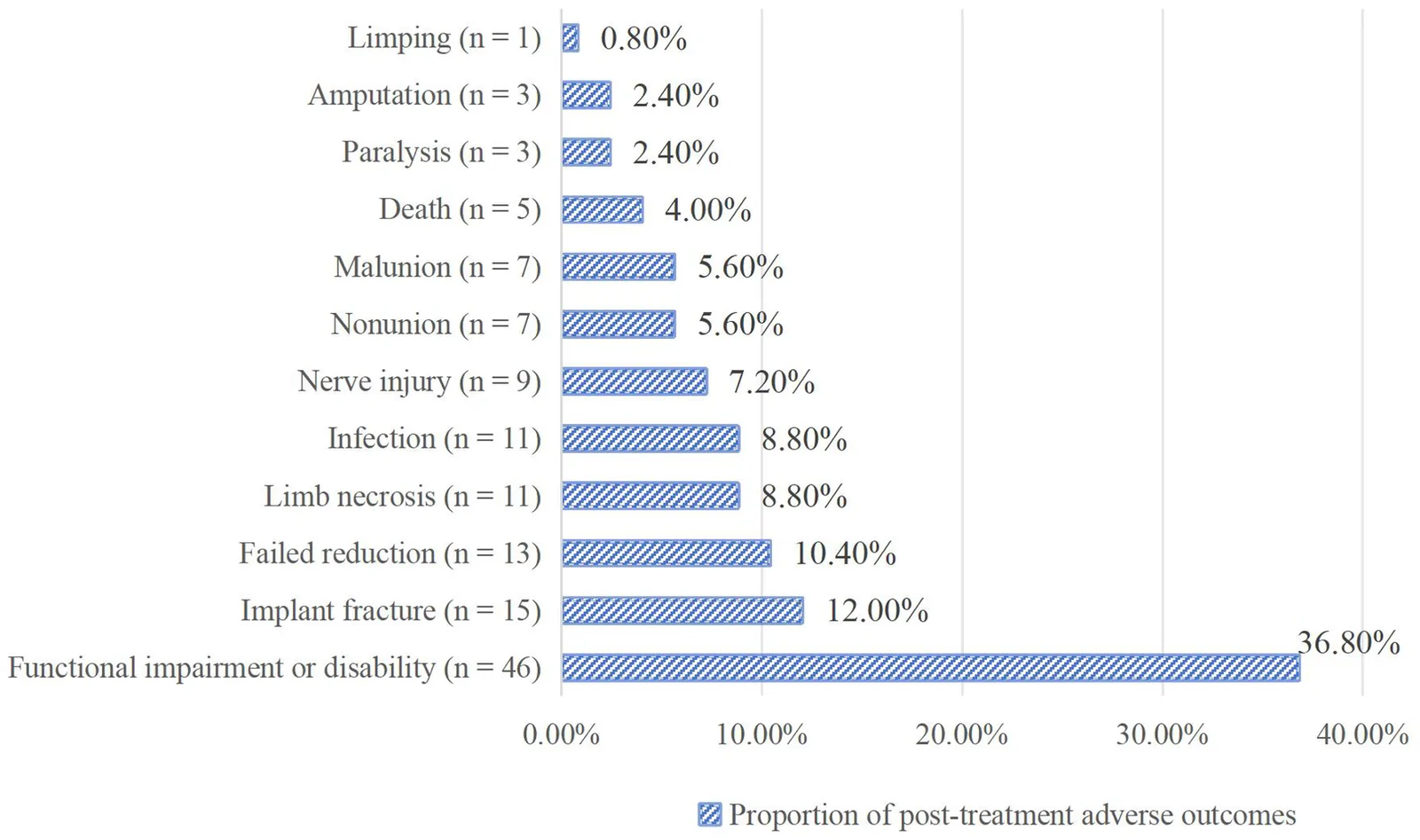
Distribution of adverse outcomes resulting from traumatic orthopedic medical malpractice.

Among the 125 patients, 93 (74.4%) developed post-malpractice disability, indicating a markedly high disability rate. The main categories of disability included functional impairment, failed reduction, amputation, infection, and nerve injury. These findings indicate that, although malpractice cases are associated with low mortality, they are accompanied by an exceptionally high incidence of long-term functional disability, imposing a substantial societal burden. This observation is consistent with previous findings reported by Luwen Shen and Fang Shi (12, 13).

### 3.4 Distribution of negligence causes in traumatic orthopedic medical malpractice

In accordance with the *2024 National Report on Medical Service and Quality Safety*, as well as the classification framework for negligent behaviors in medical malpractice established by the Office of Medical Malpractice Handling under the Shanghai Municipal Health Commission, negligence in traumatic orthopedic medical malpractice was categorized into seven types: improper surgery, violation of diagnostic and treatment protocols, missed or misdiagnosis, inadequate informed consent, insufficient observation, inadequate emergency response, and other causes. Since a single medical malpractice case often involves multiple concurrent negligent factors, this study adopted a multiple response approach for statistical analysis—i.e., when a case involved more than one negligent factor, it was counted separately in each relevant category. Consequently, the sum of frequencies across all negligent categories may exceed the total number of cases, and the cumulative percentage may be greater than 100%. In this study, the seven categories of negligent factors together accounted for 120.81% of the total, indicating that, on average, each malpractice case involved approximately 1.21 negligent factors.

As shown in Figure 3, improper surgery, missed or misdiagnosis, and violation of diagnostic and treatment protocols were identified as the three most prevalent negligence categories in traumatic orthopedic medical malpractice, accounting for 40.8%, 32.8%, and 27.2% of cases, respectively, with a combined proportion of 100.8%.

**Figure 3:**
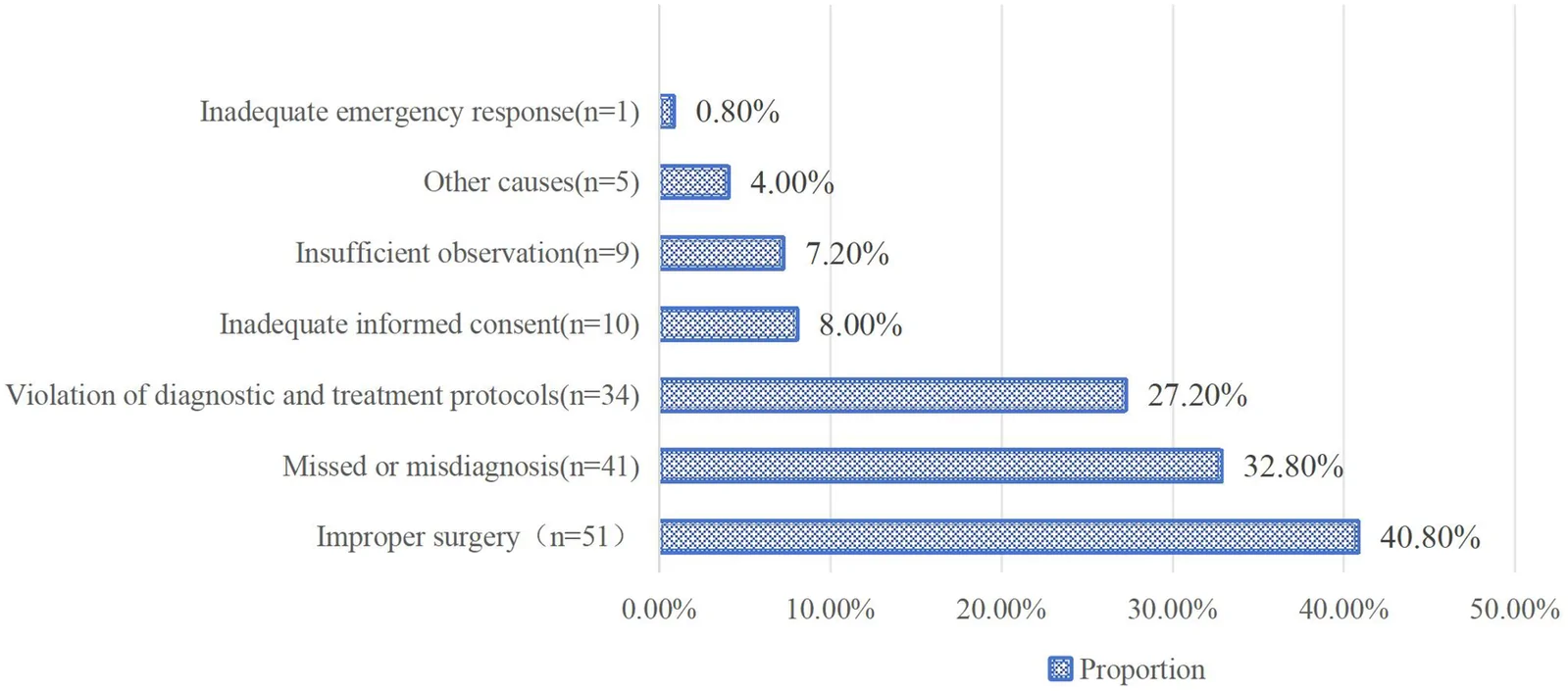
Distribution of negligence causes in traumatic orthopedic medical malpractice.

### 3.5 Comparative analysis of negligence causes across responsibility levels

#### 3.5.1 Analysis of negligence causes across different levels of medical responsibility

The level of medical responsibility in malpractice cases determines the extent of patient compensation as well as the severity of administrative or legal penalties imposed on healthcare institutions and medical personnel. In accordance with the classification criteria for responsibility levels specified in the *Regulations on the Handling of Medical Accidents*, and with reference to prior studies on the rules of causation potency, assessment principles, and legal application in medical negligence liability conducted by Wan et al. (14), Zhu (15), Zheng (16), and Yang (17), full responsibility and primary responsibility were classified into the high-responsibility group, whereas secondary responsibility and minor responsibility were categorized into the low-responsibility group. The chi-square analysis demonstrated that the proportions of improper surgery and violations of diagnostic and treatment protocols were significantly elevated in the high-responsibility group compared with the low-responsibility group (Table 4).

**Table 4:** Comparison of medical negligence behaviors between different medical responsibility level groups.

Cause	Low responsibility level group (n/%)	High responsibility level group (n/%)	*χ* ^2^	*P*
Missed or misdiagnosis			0.075	0.784
Yes	24 (33.8%)	17 (31.5%)		
No	47 (66.2%)	37 (68.5%)		
Improper surgery			8.570	0.003
Yes	21 (29.6%)	30 (55.6%)		
No	50 (70.4%)	24 (44.4%)		
Insufficient observation			0.000	1.000
Yes	5 (7.0%)	4 (7.4%)		
No	66 (93.0%)	50 (92.6%)		
Violation of diagnostic and treatment protocols			6.560	0.010
Yes	13 (18.3%)	21 (38.9%)		
No	58 (81.7%)	33 (61.1%)		
Inadequate emergency response				1.000
Yes	1 (1.4%)	0 (0%)		
No	70 (98.6%)	54 (100%)		
Inadequate informed consent			0.000	1.000
Yes	6 (8.5%)	4 (7.4%)		
No	65 (91.5%)	50 (92.6%)		
Postoperative complications			1.792	0.181
Yes	9 (12.7%)	3 (5.6%)		
No	62 (87.3%)	51 (94.4%)		
Other causes			0.000	1.000
Yes	3 (4.3%)	2 (3.8%)		
No	67 (95.7%)	51 (96.2%)		

The chi-square analysis demonstrated that the proportions of improper surgery and violations of diagnostic and treatment protocols were significantly elevated in the high-responsibility group compared with the low-responsibility group, with both differences achieving statistical significance (*P* < 0.05).

#### 3.5.2 Binary stepwise logistic regression analysis of negligence causes in traumatic orthopedic medical malpractice

To further evaluate the independent associations between individual negligence factors and high responsibility levels, a binary stepwise logistic regression analysis was performed based on the results of univariate analysis (chi-square test). The dependent variable, “degree of medical responsibility”, was dichotomized, with full responsibility and primary responsibility combined into the “high-responsibility group” (coded as 1), whereas secondary responsibility and minor responsibility were merged into the “low-responsibility group” (coded as 0). This classification approach was consistent with the grouping strategy applied in Section 3.5.1.

With the dichotomized responsibility level serving as the dependent variable, missed or misdiagnosis, improper surgery, inadequate informed consent, insufficient observation, violation of diagnostic and treatment protocols, inadequate emergency response, and other causes were included as independent variables (coding specifications are provided in Table 1). Binary stepwise logistic regression modeling was subsequently conducted. The criteria for variable entry into the model were set at *P* = 0.05, and the removal threshold was defined as *P* = 0.10.

Collinearity diagnostics revealed that the variance inflation factor (VIF) for each independent variable was less than 2, and the tolerance values were all greater than 0.7, indicating that no significant multicollinearity existed among the independent variables and that the data were suitable for logistic regression analysis (Table 5).

**Table 5:** Collinearity diagnostics among independent variables.

Variable	Tolerance	VIF
Surgical malpractice	0.796	1.257
Deviation from standard care	0.925	1.081
Missed diagnosis/misdiagnosis	0.766	1.305

Table 6 indicated that improper surgery (OR=6.078, 95% CI: 2.276–16.232, *P* < 0.001), violation of diagnostic and treatment protocols (OR=5.001, 95% CI: 1.884–13.275, *P* = 0.001), and missed or misdiagnosis (OR=2.872, 95% CI: 1.037–7.955, *P* = 0.042) were significantly positively associated with the high-responsibility group, suggesting that these factors constituted the primary negligence contributors to high-responsibility-level medical malpractice. Among these variables, improper surgery and violation of diagnostic and treatment protocols exhibited larger effect sizes (ORs), indicating a comparatively stronger association with elevated responsibility levels.

**Table 6:** Binary stepwise logistic regression of negligence causes in traumatic orthopedic diagnosis and treatment.

Variable	Regression coefficient	Standard error	Wald chi-square	*P*	OR (95% CI)
Missed or misdiagnosis	1.055	0.520	4.121	0.042	2.872 (1.037, 7.955)
Improper surgery	1.805	0.501	12.966	<0.001	6.078 (2.276, 16.232)
Violation of diagnostic and treatment protocols	1.610	0.498	10.466	0.001	5.001 (1.884, 13.275)
Constant	−1.868	0.479	15.186	<0.001	

Stepwise method was applied (entry threshold: 0.05; removal threshold: 0.10). Only statistically significant variables are presented.

Stratified analysis Table 7 further indicated that violations of diagnostic and treatment protocols were significantly associated with high responsibility levels in secondary hospitals (OR=2.94), which may be attributable to insufficient clinical competency, limited availability of advanced medical equipment, and operational pressures that contribute to deviations from standardized diagnostic and therapeutic protocols. In contrast, improper surgery was identified as the primary negligence factor associated with high responsibility levels in tertiary hospitals (OR=7.20), which may be related to the concentration of critically ill and complex cases, higher surgical volume, and an increased prevalence of postoperative complications in tertiary care settings.

**Table 7:** Binary stepwise logistic regression of negligence causes and high responsibility levels stratified by hospital level.

Hospital level	Variable entered into model	Regression coefficient	Standard error	Wald chi-square	*P*	OR (95% CI)
Secondary hospitals (*n* = 75)	Violation of diagnostic and treatment protocols	1.077	0.502	4.597	0.032	2.94 (1.10–7.86)
Tertiary hospitals (*n* = 42)	Improper surgery	1.974	0.711	7.708	0.005	7.20 (1.79–29.01)

Stepwise method was applied (entry threshold: 0.05; removal threshold: 0.10). Only statistically significant variables are presented.

#### 3.5.3 Detailed analysis of medical negligence causes based on study findings

##### 3.5.3.1 Improper surgery

Improper surgery was further stratified into preoperative, intraoperative, and postoperative categories. Approximately 28 cases (55.02%) involved preoperative deficiencies, 37 cases (72.55%) involved intraoperative deficiencies, and 16 cases (31.37%) involved postoperative deficiencies. Given that a single case may simultaneously encompass multiple categories of improper surgical conduct, the aggregated proportion across the three subcategories reached approximately 152.94%.

Preoperative negligence was primarily attributed to inadequate surgical preparation and insufficient evaluation of surgical indications. For example, in a case involving implant removal, prior operative records were not reviewed before surgery, resulting in failure to identify a retained Kirschner wire intraoperatively, which ultimately necessitated an unplanned reoperation.

Intraoperative negligence was mainly associated with inappropriate selection of surgical approach and technical errors during operative procedures. For instance, a patient admitted after a fall injury to the left upper extremity was diagnosed with a mid-shaft fracture of the left humerus. Due to inadequate intraoperative anatomical identification, inadvertent injury to the left radial nerve occurred, leading to permanent impairment of wrist function in the affected limb.

Postoperative negligence included insufficient rehabilitation guidance, delayed monitoring of postoperative conditions, and surgical site infection, among other factors. For example, following arthroplasty of the right hip with an artificial femoral head prosthesis, inadequate postoperative rehabilitation instructions resulted in prosthetic displacement, ultimately requiring revision surgery of the right hip.

Additionally, cases were identified in which multiple forms of negligence spanning the preoperative, intraoperative, and postoperative phases persisted throughout the entire perioperative period. In one representative case, a patient admitted to the emergency department following a traffic accident underwent open reduction and internal fixation for a right tibial plateau fracture. Preoperative surgical planning was insufficient; intraoperatively, the use of a single screw for fixation was deemed inappropriate; and postoperatively, cast immobilization lacked adequate rigidity, ultimately resulting in a 2 cm shortening of the right lower extremity accompanied by varus deformity.

##### 3.5.3.2 Missed or misdiagnosis

Negligence related to missed or misdiagnosis was attributed to three primary factors: substandard initial evaluation by the attending physician (*n* = 15, 36.59%), insufficient clinical diagnostic and therapeutic experience (*n* = 22, 53.66%), and failure of routine examinations to effectively detect underlying pathology (*n* = 4, 9.75%). In a representative case, a patient presented to the emergency department following trauma, and radiographic x-ray examination yielded negative findings. Therefore, no specific intervention was provided. Three days later, the patient returned with restricted mobility of the right elbow, at which time an orthopedic specialist identified a fracture of the proximal ulna on the right side and initiated appropriate treatment. Approximately 1.5 years later, the patient re-presented with valgus deformity of the right elbow, and follow-up radiographic evaluation revealed an old fracture of the proximal right ulna accompanied by dislocation of the right radial head. Over the 1.5-year follow-up period, the patient attended three medical consultations, two of which resulted in missed diagnoses.

##### 3.5.3.3 Violation of diagnostic and treatment protocols

Violations of diagnostic and treatment protocols were primarily observed in several distinct clinical contexts. First, breaches of standardized surgical and technical operation protocols were identified. In a representative case, a patient diagnosed with right knee arthritis underwent total knee arthroplasty despite markedly elevated preoperative erythrocyte sedimentation rate and C-reactive protein levels, which was subsequently followed by persistent postoperative infection and functional impairment of the knee joint. Second, failures in adherence to verification and patient identification procedures were documented. In an emergency trauma case, imaging misinterpretation resulted in a missed diagnosis, and a comminuted fracture of the left tibia and fibula was not identified until the patient's second follow-up visit. Third, noncompliance with multidisciplinary consultation protocols for complex cases was observed. In a case involving a patient who sustained a fall after alcohol consumption, early clinical signs of paraplegia were already evident in the emergency department. However, timely intervention and specialist consultation were not initiated, and the patient subsequently died due to high cervical spinal cord injury complicated by upper gastrointestinal hemorrhage. Fourth, violations of medical record documentation and management standards were documented. In 21 cases, medical records were found to be incomplete, improperly prepared, or inadequately preserved, and in some instances radiological images were missing, rendering the cases insufficient to meet evidentiary requirements in legal proceedings. Fifth, deviations from established therapeutic principles were identified. In one case involving a comminuted distal radius fracture combined with an ulnar styloid fracture, plaster immobilization was selected instead of surgical intervention, resulting in failure to achieve optimal timing for fracture reduction.

### 3.6 Receiver operating characteristic (ROC) curve analysis: discriminative performance of the model in predicting responsibility levels

To assess the discriminative performance of the binary stepwise logistic regression model in distinguishing between different levels of medical responsibility, ROC curves were constructed, and the area under the curve (AUC) was calculated (Figure 4). The ROC curve depicted the model's capacity to differentiate the high-responsibility group from the low-responsibility group across a range of threshold values.

**Figure 4:**
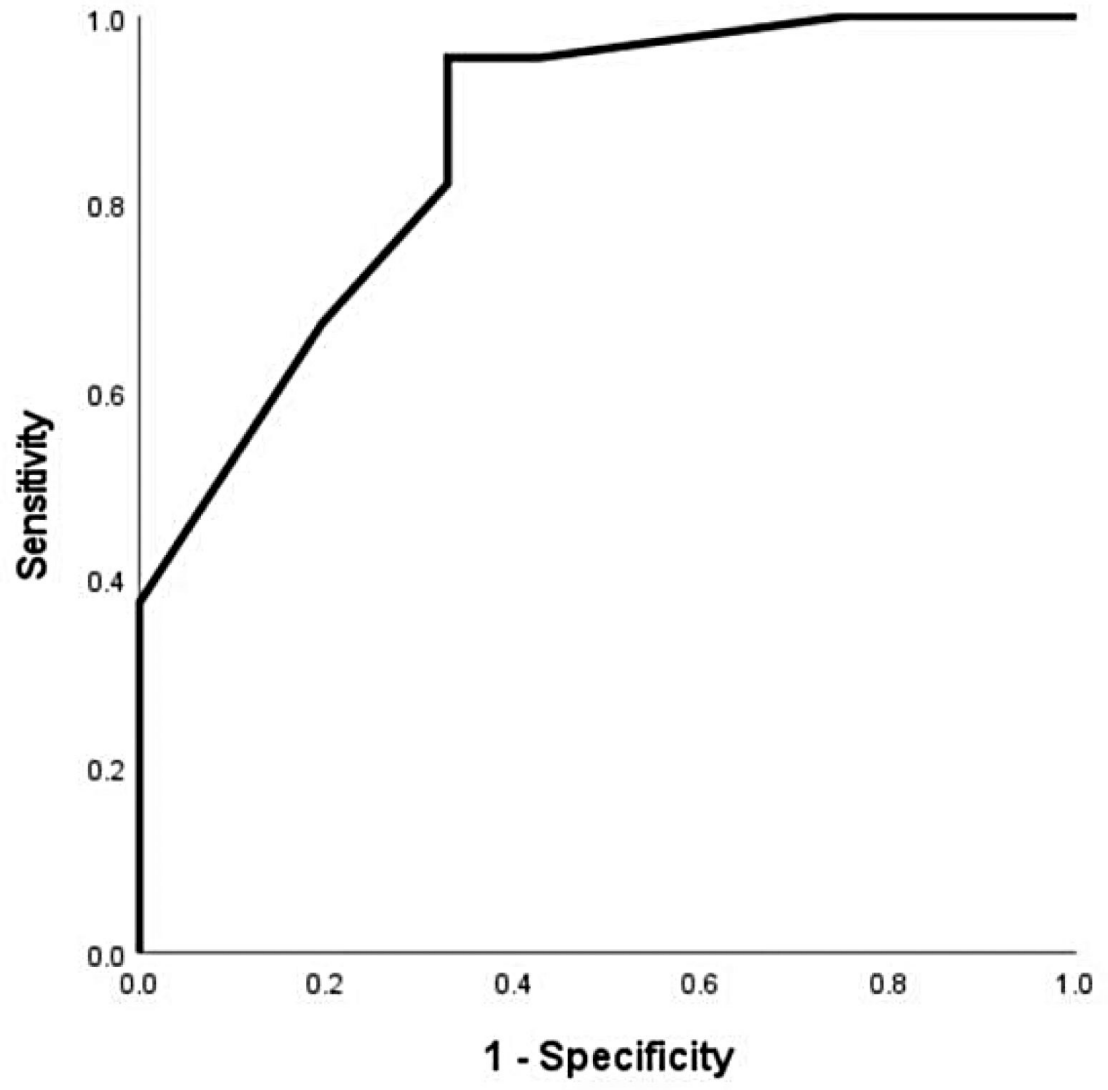
ROC curve for predicting the level of responsibility in traumatic orthopedic medical malpractice cases.

The analysis yielded an AUC of 0.727 (95% CI: 0.635–0.820), indicating satisfactory discriminatory performance. Notably, relatively high sensitivity and specificity were demonstrated by the model in identifying medical malpractice cases associated with elevated responsibility levels. These results suggest that the regression model, incorporating key variables such as surgical negligence and missed or misdiagnosis, is capable of effectively distinguishing medical malpractice cases across different responsibility strata, thereby supporting its potential applicability in clinical practice and medical jurisprudence.

## 4 Conclusions and recommendations

### 4.1 Main conclusions

Traumatic orthopedic medical malpractice in Shanghai is generally characterized by a low mortality rate, a high disability rate, a higher incidence in secondary hospitals, and a higher frequency among attending physicians and associate chief physicians. High-responsibility-level cases accounted for 45.60% of the total sample. Chi-square analysis demonstrated that the proportions of improper surgery and violations of diagnostic and treatment protocols in the high-responsibility group were significantly higher than those in the low-responsibility group (*P* < 0.05). Full-sample logistic regression analysis identified improper surgery, violations of diagnostic and treatment protocols, and missed or misdiagnosis as independent risk factors for high responsibility levels. Stratified regression further indicated that, in secondary hospitals, violations of diagnostic and treatment protocols represented the primary negligence factor, whereas in tertiary hospitals, improper surgery was the predominant determinant. This variation suggests that the principal negligence drivers of high-responsibility-level malpractice differ across hospital tiers.

International comparisons yielded the following observations (19–21): in traumatic orthopedic data from the UK NHS, the most frequently reported claim types included mismanagement, diagnostic errors, and perioperative complications; in India, 53% of traumatic orthopedic complaints were attributable to surgical issues, with small- and medium-sized hospitals exhibiting the highest surgery-related complaint rates; litigation data from the United States indicated that diagnostic errors and procedural errors were among the most commonly cited causes; in Australia, diagnostic errors were predominant in traumatic orthopedic malpractice cases; and data from Iran indicated that 53% of cases were attributable to technical errors during surgical procedures (18). The findings from Shanghai in the present study identified improper surgery, violations of diagnostic and treatment protocols, and missed or misdiagnosis as the three leading causative factors. Collectively, deficiencies in diagnostic processes, surgical execution, and adherence to standardized diagnostic and treatment protocols appear to represent consistently prevalent sources of medical negligence across different healthcare systems internationally.

### 4.2 Recommendations

#### 4.2.1 Recommendations regarding improper surgery

First, preoperative discussion protocols should be strictly followed, necessary consultations should be completed, surgical indications and contraindications should be rigorously evaluated, and preoperative communication with patients and their families should be strengthened, with signed documentation retained for the record. Second, intraoperative procedures should strictly adhere to surgical operating protocols, and any problems encountered should be promptly reported for guidance. Third, vigilant postoperative monitoring of the patient's general condition should be maintained, with particular attention to hemorrhage, infection, and vascular perfusion, and postoperative care and rehabilitation guidance should be reinforced. Fourth, technical support and guidance from tertiary hospitals to secondary hospitals should be strengthened, and the allocation of large medical equipment in secondary hospitals should be improved. Fifth, the hospital tiering management system and the surgical classification management system should be strictly implemented.

#### 4.2.2 Recommendations regarding missed or misdiagnosis

First, for critically ill patients, direct transport to tertiary hospitals by 120 emergency services should be ensured, and hospitalization should be recommended when necessary to compensate for the limitations of initial assessment. Second, the first-contact responsibility system should be enforced, standardized management of diagnosis and treatment by first-contact physicians should be strengthened, and timely reporting and consultation should be pursued when experience is insufficient. Third, the medical ethics, sense of responsibility, theoretical knowledge, and technical skills of medical personnel with approximately 10 years of work experience should be enhanced. Fourth, virtual surgical simulation systems should be utilized when necessary to assist physicians in familiarizing themselves with local anatomy and formulating preoperative plans.

#### 4.2.3 Recommendations regarding violation of diagnostic and treatment protocols

First, strict implementation of the 18 core institutional systems for quality and patient safety should be ensured. Second, medical record documentation should be standardized to guarantee that records are objective, authentic, and complete. Third, laws, regulations, diagnostic and treatment protocols, and institutional rules should be strictly complied with. Fourth, physician–patient communication should be enhanced, the duty of disclosure should be diligently fulfilled, and patients’ right to informed consent should be safeguarded. Fifth, the clinical competency of personnel in secondary hospitals should be improved and the level of medical equipment should be upgraded, thereby reducing protocol violations at their source. Sixth, participation of patients and their families should be encouraged, and the shared decision-making mechanism between physicians and patients should be strengthened.

#### 4.2.4 Limitations

First, although the sample size of 125 cases is relatively substantial compared to similar studies, all cases were concentrated in Shanghai. As one of the most developed cities in China in terms of healthcare resources, Shanghai's hospital tiering structure, medical personnel training system, and healthcare quality management level may differ from those in other regions of the country. Therefore, the generalizability of the findings is limited.

Second, the cases in this study were derived from Medical Association technical appraisal conclusions. Although these conclusions are professionally and administratively authoritative, we were unable to obtain complete patient demographic information (e.g., income, education level, occupation, etc.) or detailed intraoperative and perioperative clinical data (e.g., surgical duration, anesthesia method, intraoperative monitoring parameters, etc.). The absence of such information limited our ability to conduct in-depth quantitative analyses of these dimensions.

Third, in the stratified regression analyses, although the sample sizes for secondary hospitals (*n* = 75) and tertiary hospitals (*n* = 42) generally met the basic requirements for stepwise regression, statistical power remained limited. Other negligence factors that were not retained in the regression models may have exerted weak effects or exhibited multicollinearity within the present dataset, and thus warrant further validation in future multi-center studies with larger sample sizes.

Despite these limitations, the dataset analyzed in this study represents one of the more comprehensive investigations of traumatic orthopedic medical malpractice in China. The findings provide actionable evidence to support improvements in clinical diagnostic and therapeutic standards, reduction of medical negligence, and enhancement of patient safety.

## Data Availability

The original contributions presented in the study are included in the article/Supplementary Material, further inquiries can be directed to the corresponding author/s.
