# Supplementary material for: Analysis of causative factors and preventive strategies for traumatic orthopedic medical malpractice in Shanghai: a high-responsibility perspective
Source: Front Surg. 2026 Oct 30;13:1930290. doi: 10.3389/fsurg.2026.1930290 (PMC13759797; doi:10.3389/fsurg.2026.1930290)
Supplement: Supplementary file 1 [file Table1.docx]

**Appendix**

**1. Definition and constitutive elements of medical malpractice under the *Regulations on the Handling of Medical Accidents***‌

Medical malpractice refers to adverse events in which a medical institution and its personnel, during the provision of medical services, are found to have violated laws and administrative regulations governing healthcare management, departmental rules, or standardized protocols for diagnosis, treatment, and nursing care, thereby negligently causing harm to patients.

**1.1 The constitutive elements of medical malpractice shall be simultaneously satisfied as follows:**

1.1.1 The subject is a medical institution and its medical personnel;

1.1.2 The malpractice occurs in the course of medical activities;

1.1.3 The medical conduct giving rise to the malpractice is unlawful;

1.1.4 The medical institution and its medical personnel are subjectively at fault;

1.1.5 A causal relationship exists between the negligent conduct and the resulting harm.

1.2 Based on the degree of personal injury caused to the patient, medical malpractice is classified into four grades:

1.2.1 Grade I medical malpractice: resulting in the patient’s death, severe disability, or persistent vegetative state;

1.2.2 Grade II medical malpractice: resulting in moderate disability or serious functional impairment due to organ or tissue damage;

1.2.3 Grade III medical malpractice: resulting in mild disability or general functional impairment due to organ or tissue damage;

1.2.4 Grade IV medical malpractice: resulting in other consequences constituting apparent personal injury to the patient.

**1.3 Circumstances not constituting medical malpractice**

The *Regulations on the Handling of Medical Accidents* stipulate that none of the following circumstances shall be deemed medical malpractice:

1.3.1 Adverse outcomes arising from emergency medical measures taken to save the life of a critically ill patient;

1.3.2 Medical accidents occurring in the course of medical activities due to unusual disease conditions or special physical constitution of the patient;

1.3.3 Adverse outcomes resulting from transfusion-transmitted infection without fault;

1.3.4 Adverse outcomes caused by delayed diagnosis or treatment attributable to the patient’s own reasons;

1.3.5 Adverse outcomes caused by force majeure.

**2. Factors relevant to compensation amounts in medical malpractice**

2.1 The grade of the medical malpractice;

2.2 The degree of responsibility attributable to the negligent medical conduct in producing the harmful outcome;

2.3 The relationship between the harmful outcome of the medical malpractice and the patient’s pre-existing medical condition.

### **3 Medical malpractice technical appraisal**

3.1 Medical malpractice technical appraisal is organized and conducted by medical associations at various levels. Medical associations are academic social organizations composed of medical science and technology professionals.

**3.2 Eligibility criteria for appraisal experts:**

3.2.1 Possessing sound professional competence and ethical conduct in practice;

3.2.2 Employed by a medical and health institution or a medical education or research institution, and having held a senior professional and technical position in the relevant specialty for at least three years;

3.2.3 Being in a state of health sufficient to perform medical malpractice technical appraisal duties.

**3.3 Selection of appraisal experts:**

In accordance with Article 17 of the *Provisional Measures on Technical Appraisal of Medical Accidents*, experts eligible for selection shall be assigned randomized identification numbers by the medical association. Under its supervision, both parties shall randomly select an equal number of experts, and the final expert shall be randomly determined by the medical association. Each party shall additionally select one alternate expert using the same procedure. In cases involving determination of cause of death or disability grading, a forensic physician shall be separately selected by each party to participate in the appraisal panel. A recusal system shall also be implemented to ensure procedural impartiality.

### **3.4 The medical malpractice technical appraisal report shall include the following principal contents:**

### 3.4.1 Whether the medical conduct violates laws and administrative regulations governing healthcare management, departmental rules, and standards and routines for diagnosis, treatment, and nursing care;

### 3.4.2 Whether a causal relationship exists between the negligent medical conduct and the personal injury outcome;

### 3.4.3 The degree of responsibility attributable to the negligent medical conduct in producing the harmful outcome of the medical malpractice;

### 3.4.4 The grade of the medical malpractice;

### 3.4.5 Medical and nursing recommendations for the patient involved in the medical malpractice.

### **3.5 Legal effect of appraisal conclusions**

Appraisal conclusions in medical malpractice cases shall serve primarily as the basis for administrative decision-making by health authorities and may be directly admitted as evidence in judicial proceedings.

**4 Hospital Classification System in China**

Hospitals in China are classified into three tiers based on indicators such as institutional scale, research orientation, professional and technical capacity, and availability of medical equipment. These include tertiary Grade A hospitals, tertiary Grade B hospitals, secondary Grade A hospitals, secondary Grade B hospitals, and primary hospitals (community health service centers), with tertiary Grade A hospitals representing the highest level of care. According to statistical data from the Shanghai Municipal Health Commission, approximately 30%–50% of patients in Shanghai hospitals originate from outside the city, with this proportion exceeding 60% in some top-tier tertiary Grade A institutions.

**5 Disciplinary positioning and definition of traumatic orthopedics**

Within the Chinese academic discipline classification system:

5.1 First-level discipline: Clinical Medicine

5.2 Second-level discipline: Surgery

5.3 Third-level discipline: Orthopedics

5.4 Subspecialties: Traumatic Orthopedics, Spinal Surgery, Joint Surgery, Foot and Ankle Surgery, etc.

Traumatic orthopedics is a major subspecialty of orthopedics focusing on the diagnosis and management of acute injuries to bones, joints, and associated soft tissues caused by trauma. Its core scope includes fractures, joint dislocations, and open injuries involving bone structures, as well as emergency management and functional restoration of complex trauma such as traffic accidents and falls from height. All cases included in this study were strictly limited to this subspecialty.

**6 Physician title system in China**

Physician titles in China are obtained through a structured and standardized evaluation system. The hierarchy progresses from resident physician to attending physician, associate chief physician, and ultimately chief physician, with promotions typically occurring at approximately five-year intervals. Physician titles are independent of hospital classification. Physicians holding the same title are considered to possess comparable qualifications and clinical competence regardless of institutional tier.

**7 Eighteen core medical quality and safety systems**

7.1 First-contact physician responsibility system;

7.2 Three-tier physician ward round system;

7.3 Consultation system;

7.4 Hierarchical nursing care system;

7.5 On-call duty and handover system;

7.6 Difficult case discussion system;

7.7 Critical patient rescue system;

7.8 Pre-operative discussion system;

7.9 Death case discussion system;

7.10 Medical verification system;

7.11 Surgical safety checklist system;

7.12 Surgical grading management system;

7.13 New technology and new project access system;

7.14 Critical value reporting system;

7.15 Medical record management system;

7.16 Clinical blood use review system;

7.17 Graded antimicrobial drug management system;

7.18 Information security management system.
